# Effects of CeO_2_ Content on Friction and Wear Properties of SiCp/Al-Si Composite Prepared by Powder Metallurgy

**DOI:** 10.3390/ma13204547

**Published:** 2020-10-13

**Authors:** Bin Yang, Aiqin Wang, Kunding Liu, Chenlu Liu, Jingpei Xie, Guangxin Wang, Shizhong Wei

**Affiliations:** 1 Materials Science and Engineering School, Henan University of Science and Technology, Luoyang 471023, China; aiqin_wang@haust.edu.cn (A.W.); liukunding@haust.edu.cn (K.L.); liuchenlu@haust.edu.cn (C.L.); xiejp@haust.edu.cn (J.X.); wgx58@haust.edu.cn (G.W.); wsz@haust.edu.cn (S.W.); 2 Provincial and Ministerial Co-Construction of Collaborative Innovation Center for Non-Ferrous Metal New Materials and Advanced Processing Technology, Luoyang 471023, China

**Keywords:** powder metallurgy, particle reinforced composites, rare earth element, mechanical properties, friction and wear properties

## Abstract

SiCp/Al-Si composites with different CeO_2_ contents were prepared by a powder metallurgy method. The effect of CeO_2_ content on mechanical properties, friction and wear properties of the composites was studied. The results show that with the increase in CeO_2_ content from 0 to 1.8 wt%, the density, hardness, friction coefficient of the composites first increases and then decreases, the coefficient of thermal expansion (CTE) and wear rate of the composites first decreases and then increases. When the content of CeO_2_ was 0.6 wt%, the density and hardness of the composite reached the maximum value of 98.54% and 113.7 HBW, respectively, the CTE of the composite reached the minimum value of 11.1 × 10^−6^ K^−1^, the friction coefficient and wear rate of the composite reached the maximum value of 0.32 and the minimum value of 1.02 mg/m, respectively. CeO_2_ has little effect on the wear mechanism of composites, and the wear mechanism of composites with different CeO_2_ content is mainly abrasive wear under the load of 550 N. Compared with the content of CeO_2_, load has a great influence on the wear properties of the composites. The wear mechanism of the composites is mainly oxidation wear and abrasive wear under low load. With the increase in load, the wear degree of abrasive particles is aggravated, and adhesive wear occurs under higher load.

## 1. Introduction

Particulates reinforced aluminum matrix composites (PRAMCs), have been well recognized and progressively improved because of their high strength–weight ratio, good wear resistance and high thermal stability, etc. [1,2,3]. Among these superior properties, the excellent wear resistance of PRAMCs has increased attention towards tribological applications, especially, hypereutectic Al-Si alloy matrixes with 13–20 wt% Si, have been widely used for applications in automobile components, such as engine blocks, cylinder, heads, brake rotors and pistons, due to the merits of their light weight, high specific strength and excellent wear resistance [4,5,6,7]. So far various commonly used reinforcement particulates have been used, such as TiB_2_ [8], B_4_C [9], SiO_2_ [10], Al_2_O_3_ [11], TiC [12], Si_3_N_4_ [13] and SiC [14]. Among them, SiC is considered to be most common form of particulate reinforcement because it can not only increases the tensile strength, hardness and density but also improves the wear resistance of aluminum matrix composites. Compared with the traditional liquid metallurgy methods such as stirring casting, squeeze casting and compo casting, the distribution of reinforcing particles is uniform, and the content is easily adjustable when the composite is prepared by powder metallurgy [15,16,17]. Moreover, due to the relatively low sintering temperature, the reaction of the matrix and the reinforcing particles in the preparation process has been avoided. However, during the sintering process of SiCp/hypereutectic Al-Si composites, a large amount of coarse polygonal precipitated Si is produced, which is brittle and seriously splits the matrix. As a consequence, the mechanical properties and machinability of the composites are significantly reduced. Therefore, it is of great importance to control the particle size, morphology and distribution of precipitated Si in matrix for fabricating SiCp/hypereutectic Al-Si composites with superior performance.

Rare earth (REs) elements have been increasingly used as reinforcement materials in various aluminum matrix composites due to their high strength at room temperature, good thermal conductivity and mechanical properties [18,19]. Most studies indicate that the presence of rare earth in the composites leads to microstructural refinement, reduced grain size, uniform alloy elements, and enhanced tribo-mechanical properties. Li et al. [20] found that the addition of 1.0 wt% Ce significantly refined the primary Si and transferred the morphology from coarse irregular to fine blocky. Kilicaslan et al. [19] observed that rare earth Sc refined the primary Si without dramatically changing the morphology in Al-20Si alloys. Wu et al. [21] fabricated Ti/Al_2_O_3_ composites with a different volume content of CeO_2_ via vacuum hot-pressing sintering and found that the addition of CeO_2_ could significantly improve the microhardness, flexural strength and fracture toughness. Pramod [22] and Lohar [23] have reported that a minor addition of Sc in TiB_2_ reinforced Al-matrix composites can reduce the grain size and improve the mechanical properties of the composites by modifying the eutectic Si or the generation of new precipitates. Wang et al. [24] found that the addition of CeO_2_ can significantly refine the microstructure and improve the wear resistance of SiCp/Al-Si composites, but there is lack of in-depth analyses into the effects of CeO_2_ on grain refinement mechanism and wear mechanism. Although intensive research has been conducted on the effects of rare earth element’s addition on the grain size and mechanical properties of PRAMCs, most of this research is focused on composites prepared by liquid metallurgy methods. Thus, little attention has been paid to the effects of rare earth elements on the wear performance of SiCp/hypereutectic Al-Si composites.

In the present work, SiCp/Al-Si composites with different CeO_2_ contents were fabricated by a powder metallurgy method. The aim of the present study was to reveal the effect of CeO_2_ content on microstructure, friction and wear properties. The results can provide a new approach to designing wear-resistant PRAMCs and other hard particulate reinforced light alloy composites.

## 2. Experimental Process

### 2.1. Materials and Sample Preparation

The Al-19Si-1.5Cu-0.6Mg alloy powder with average particle size of 10–15 μm, prepared by the gas-atomization method was used as the matrix material, and the density of the Al matrix powder was 2.65 g/cm^3^. SiCp with an average particle size of 10μm was selected as a reinforcement material, with a mass fraction of 20 wt%. High purity nano CeO_2_ powder was added as the reinforcement, with a mass fraction of, respectively, 0 wt%, 0.15 wt%, 0.3 wt%, 0.6 wt%, 1.2 wt%, 1.8 wt%.

In order to improve the properties of raw material powders, the matrix powder and reinforcement powder were pretreated. The pretreatment process of the matrix powder is as follows: firstly, the powder was cleaned with alcohol, then ultrasonic cleaned in distilled water, finally, the matrix powder was dried in a vacuum drying oven at 70 °C. The pretreatment method of SiCp powder is as follows: firstly, the SiCp powder is calcined at 1000 °C for 3 h, and then slowly cooled to room temperature, then ultrasonic cleaned in distilled water, and finally dried in a vacuum drying oven at 70 °C. The composite powder was prepared by ball milling the above powder with for 4 h at ambient temperature under argon atmosphere. Alcohol was added as a dispersant in the mixing process, the mass ratio of alcohol to powder was 1:8, the mass ratio of ball to powder was 2:1 and the speed of ball mill was 250 r/min. After ball milling, the mixed powder was dried in a vacuum drying oven at 70 °C.

**Figure 1:**
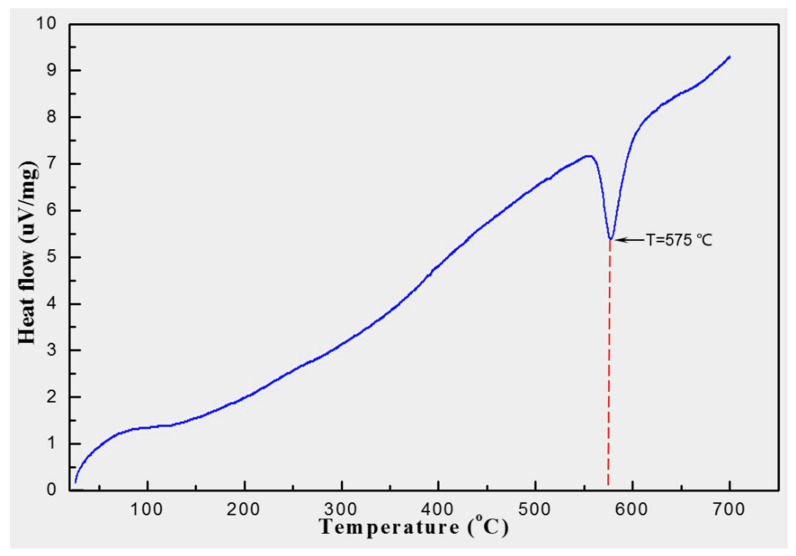
Differential scanning calorimetry (DSC) curve of mixed powder.

The thermal stability of the mixed powder was analyzed by a differential scanning calorimetry analyzer (DSC, NETZSCH, STA409PC, Selb, Germany). The test conditions are as follows: the starting temperature was 25 °C, the ending temperature was 700 °C, the heating rate was 20 °C/min and the test process was carried out under a nitrogen atmosphere. Figure 1 shows the DSC curve of mixed powder. It can be concluded that there is no obvious endothermic phenomenon when the heating temperature is lower than 550 °C, with the continuous increase in heating temperature, the endothermic rate is obviously accelerated and an endothermic peak appears. When the heating temperature rises to 575 °C, the endothermic peak reaches the bottom, and the endothermic peak ends at 608 °C. Therefore, the suitable sintering temperature of the mixed powder is around 550 °C.

**Figure 2:**
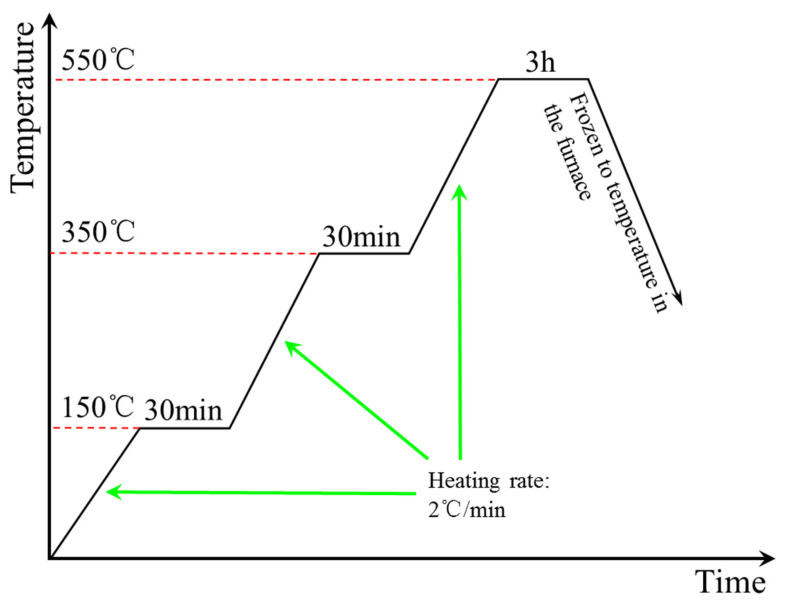
Sintering process curve of composite powder.

The mixed powder was put into a pressing mould and cold-rolled to a diameter of 78 mm, length of 48 mm billet with 500 MPa pressure in a hydraulic machine. Then the billets were heated in a tube furnace with sintering temperature of 550 °C and sintering time of 3 h (technology curve of sintering is shown in Figure 2, protective gas is N_2_.). Finally, the billets were cut into various standard test samples by wire cutting.

### 2.2. Microstructural Characterization and Performances Test

The microstructure of the composites was characterized by a scanning electron microscopy (SEM, JEOL, JSM-5600LV, Tokyo, Japan) with energy dispersive spectroscopy (EDS, Kevex), and transmission electron microscope (TEM, JEOL, JEM-2100, Tokyo, Japan). The average diameter and shape factor of Si particles precipitated from the composites with different CeO_2_ content was measured by Image Pro Plus software v6.0. This software generates histograms of precipitated Si particles from the SEM image and quantifies the diameter/perimeter/area of precipitated Si through line profile analysis which provides the average grain size and shape factor of all precipitated Si particles. The hardness of the prepared materials was measured by a Brinell hardness tester (HB-3000B, Beijing, China). The CTE of the composites was measured by a thermal expansion coefficient tester (PCY-3, Beijing, China). The test temperature was 20 °C to 100 °C, the heating rate was 100 °C/s. The theoretical density of the composite is calculated by the following formula:

(1) pct=mAlpAl+mSiCpSiC+mCeO2pCeO2−1

In Equation (1), *p_ct_* is the theoretical density of the composite, g/cm^3^; *m_Al_/m_SiC_/m_CeO2_* is the mass fraction of Al matrix powder, SiCp powder and CeO_2_ powder, respectively, wt%; *p_Al_/p_SiC_/p_CeO2_* is the theoretical density of Al matrix powder, SiCp powder and CeO2 powder, respectively, g/cm^3^. The actual density of the composite was measured by Archimedes drainage method. According to the measured actual density and the calculated theoretical density, the relative density of the composite can be calculated.

**Figure 3:**
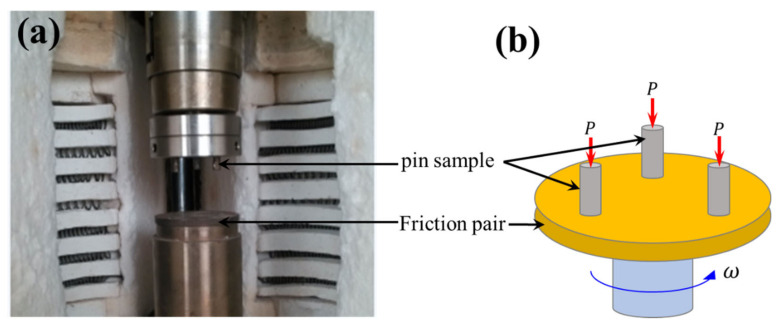
Basic schematic of the MMU-5GA abrasion tester: (**a**) Physical photos; (**b**) Wear schematic diagram.

The friction and wear tests were carried out on the self-made MMU-5GA abrasion tester (see Figure 3). The friction pair material was ASTM-1045 steel, the positive force was 500–700 N, the relative sliding speed was 10 m/s, and the test time was 5 min. Before the test, the pin samples and friction pair were polished with 800 mesh sandpaper and cleaned with alcohol. Each experiment was repeated three times, and the results were averaged. The frictional curves with sliding time were recorded by a computer procedure. The wear surface was observed by a scanning electron microscopy (SEM, JEOL, JSM-5600LV, Tokyo, Japan) with energy dispersive spectroscopy (EDS, Kevex).

## 3. Results and Discussion

### 3.1. Microstructure and Properties of Composites

**Figure 4:**
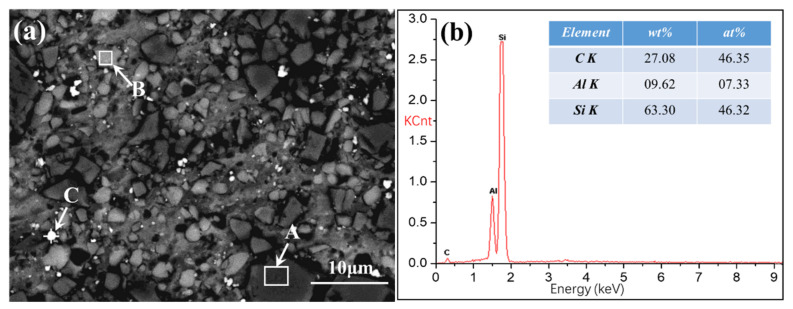

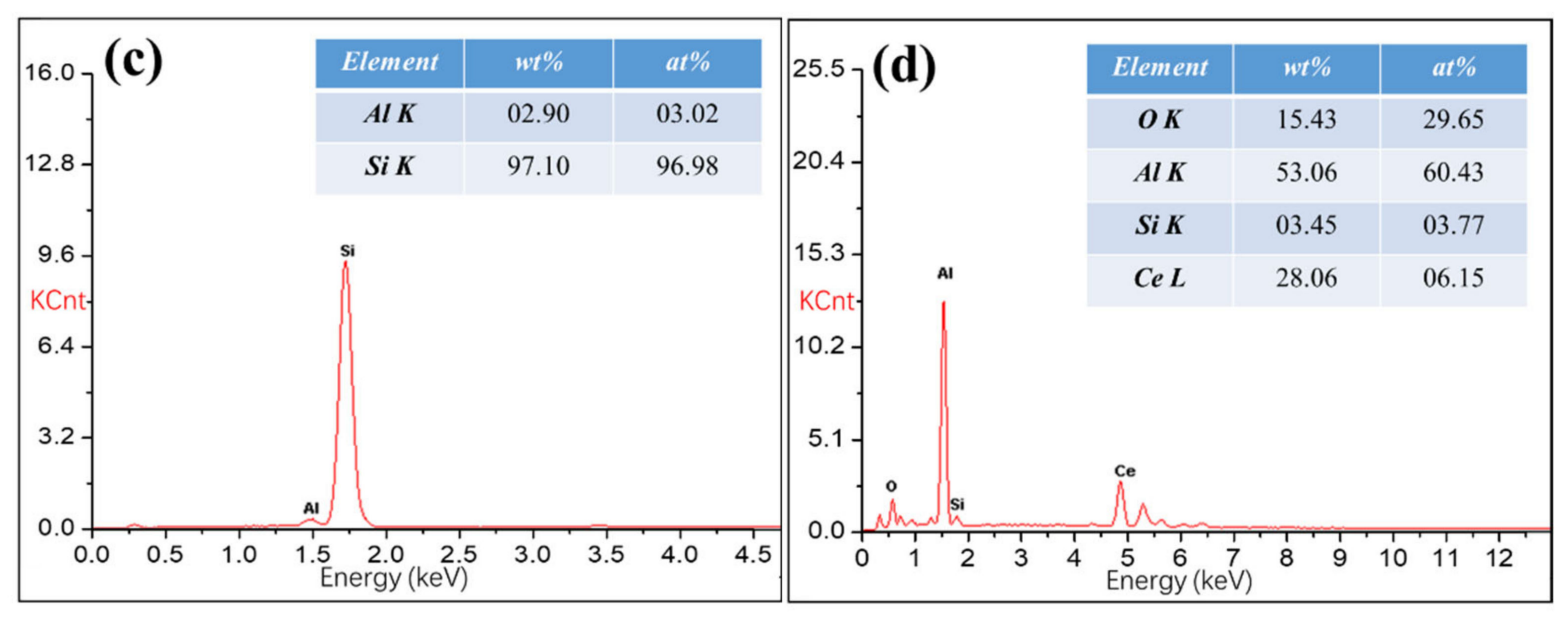
The scanning electron microscope (SEM) and energy dispersive spectrometer (EDS) of SiCp/Al-Si Composites with 0.6 wt% CeO_2_: (**a**) the SEM of diagram organization; (**b**) the energy spectrum diagram of region A; (**c**) the energy spectrum diagram of region B; (**d**) the energy spectrum diagram of point C.

Figure 4 shows the microstructure and energy spectra of SiCp/Al-Si Composites with 0.6 wt% CeO_2_ content. Figure 4a shows the SEM picture of the composite. Figure 4b shows the energy spectra diagram of region A in Figure 4a, Figure 4c shows the energy spectra diagram of region B in Figure 4a, and Figure 4d shows the energy spectra diagram of point C in Figure 4a. It can be seen from the figure that the dark gray particle phase in Figure 4a (region A) is SiC, the light gray particle phase (region B) is precipitated Si, and the white particle phase (region C) is CeO_2_. The distribution of three kinds of particles is uniform, and there are a few holes in the composite, and the material is relatively dense.

**Figure 5:**
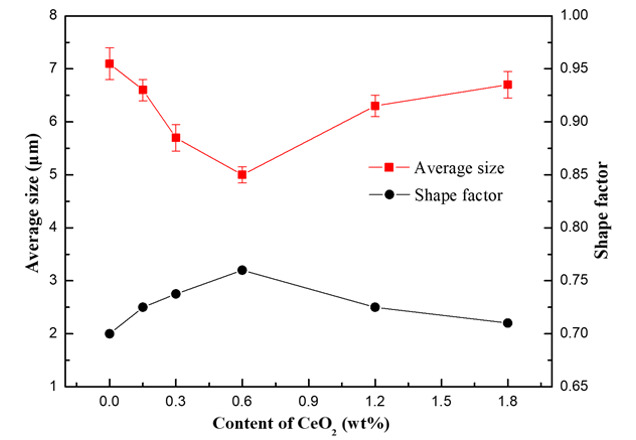
The average diameter and shape factor of Si particles precipitated in the composites with different CeO_2_ contents.

The average diameter and shape factor of Si particles precipitated in the composites with different CeO_2_ contents is shown in Figure 5. It can be seen from Figure 5 that the average size of precipitated Si particles in the composite without CeO_2_ is the largest, and the appropriate amount of CeO_2_ can significantly refine the precipitated Si. When the content of CeO_2_ is less than 0.6 wt%, the average diameter of precipitated Si decreases with the increase in CeO_2_ content, when the content of CeO_2_ is more than 0.6 wt%, the average diameter of precipitated Si increases with the increase in CeO_2_ content. The average diameter of the precipitated Si in the composite reaches the minimum when the content of CeO_2_ is 0.6 wt%. The difference of shape factor of precipitated Si in the composites with different CeO_2_ content is relatively small, which indicates that CeO_2_ content has little effect on the morphology of precipitated silicon particles.

**Figure 6:**
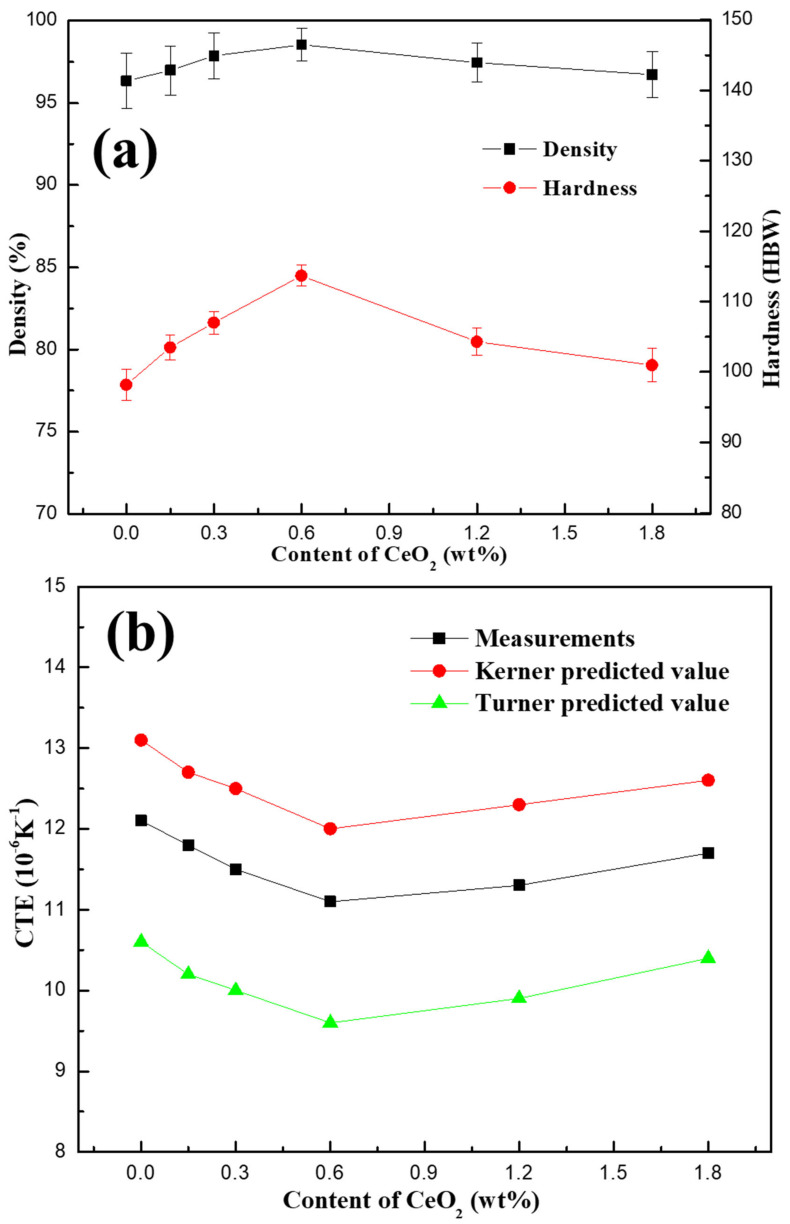
The density, Brinell hardness and coefficient of thermal expansion (CTE) of SiCp/Al-Si Composites with different CeO_2_ contents: (**a**) density and hardness; (**b**) CTE.

Figure 6a shows the density and Brinell hardness of SiCp/Al-Si Composites with different CeO_2_ contents. It can be seen that when the CeO_2_ content is less than 0.6 wt%, the density and hardness of the composites gradually increases with the increase in CeO_2_ content, and when the CeO_2_ content is greater than 0.6 wt%, the density and hardness of the composite gradually decreases with the increase in CeO_2_ content. The density and hardness of the composite reached the maximum value of 98.54% and 113.7 HBW, respectively, when the content of CeO_2_ is 0.6 wt%.

When the content of CeO_2_ is less than 0.6 wt%, the average size of the precipitated Si particles in the composites decreases with the increase in CeO_2_ content, and the effective carriers in the unit area of the composite gradually increase, the bonding state of fine precipitated Si particles with the matrix is relatively close, which reduces the porosity of the material. Therefore, the density and hardness of the composites gradually increase with the increase in CeO_2_ content. When the CeO_2_ content exceeds 0.6 wt%, the average size of the precipitated Si particles in the composites begins to increase gradually, which worsens the bonding state between the precipitated Si particles and the matrix, resulting in the increase in pores. At the same time, the effective carrier in unit area of the composite decreases, so the density and hardness of the composites gradually decrease.

Figure 6b shows the CTE of SiCp/Al-Si composites with different CeO_2_ contents obtained by measurement and Turner/Kerner theoretical calculation models [25,26]. It can be seen that the change in the trend of CTE obtained by measurement and theoretical calculation is consistent with the increase in CeO_2_ content, the CTE of the composites first decreases and then increases with the increase in CeO_2_ content. When the CeO_2_ content is 0.6 wt%, the CTE obtained by measurement of the composite reaches the lowest value of 11.1 × 10^−6^ K^−1^. It is remarkable that the measured value of CTE is larger than that calculated by the Turner model but smaller than that calculated by Kerner model. This is because the Turner model assumes that the stress in the composite is uniform static stress, while the Kerner model assumes that the reinforcement phase of the composite is spherical particles. However, there are complex non-uniform stresses in the composites, the SiC particles and precipitated Si particles in the composites are also irregular particles. Therefore, there is a certain deviation between the calculated values of the two models and the measured values.

It is well known that the thermal expansion properties of SiCp/Al Si composites are mainly affected by the thermal expansion properties of Al matrix, SiCp and precipitated Si particles, and also related to the density of the composites. According to Figure 6a, when the CeO_2_ content is too high or too low, the density of the composite is low, the microstructure is relatively loose, and the pores are increased. Therefore, there is more space for atoms to vibrate when the composite was heated, thus reducing the thermal expansion coefficient and improving the thermal stability of the composite.

### 3.2. Effect of CeO_2_ Additions on Friction and Wear Properties of SiCp/Al-Si Composites

**Figure 7:**
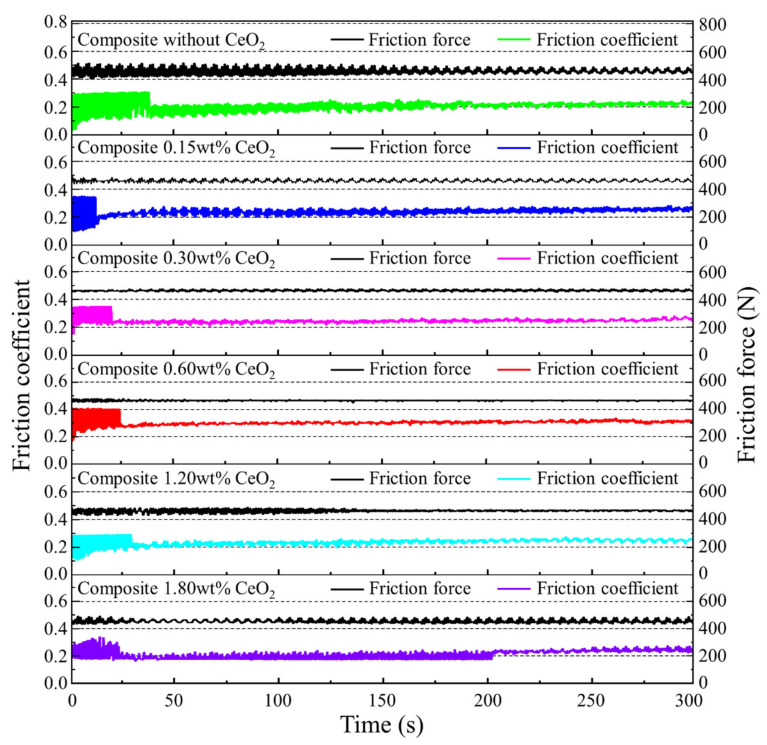
The curve of dynamic current-carrying friction coefficient and friction force of SiCp/Al-Si Composites with different CeO_2_ contents.

Figure 7 shows the change in dynamic friction coefficient and friction force of SiCp/Al-Si composites with different CeO_2_ content under 550 N load at room temperature. It can be seen from the diagram that the friction coefficient fluctuated obviously in the early stage of each test process. However, it tended to be stable after a period of time, which shows that each wear sample entered a relatively stable wear stage after a certain running-in period. In addition, with the increase in CeO_2_ content, the fluctuation amplitude of the friction coefficient and friction force first decreases and then increases. When the content of CeO_2_ is 0.6 wt%, the fluctuation amplitude of the friction coefficient and friction force is the smallest. The main reason for this phenomenon may be that when the CeO_2_ content is 0.6 wt%, the average size of the precipitated Si phase in the composite is the smallest and the most round. These small Si precipitated phases can provide a better lubrication effect for the wear surface. There is only a little visible vibration and noise for the composites with different CeO_2_ content during the whole sliding process.

**Figure 8:**
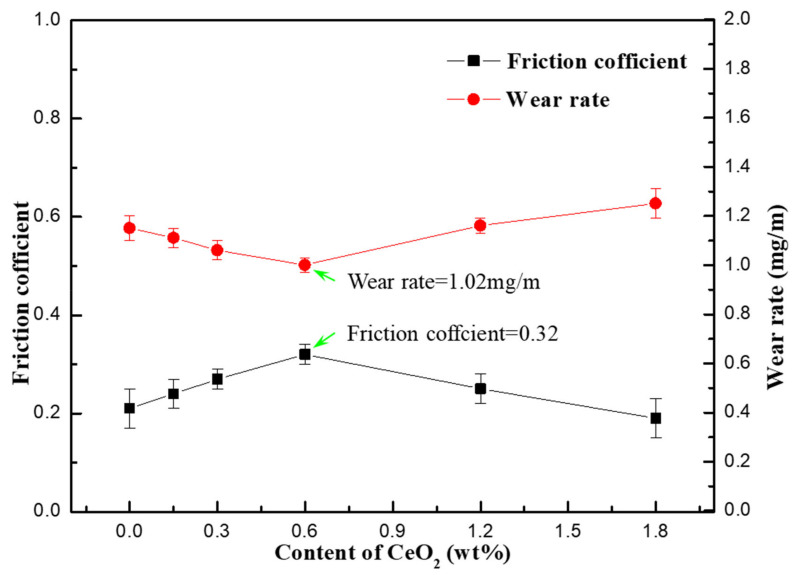
The curves of the friction coefficient and wear rate of SiCp/Al-Si composite with different CeO_2_ contents.

Figure 8 shows the friction and wear properties of SiCp/Al-Si composites with different CeO_2_ content. It can be seen from Figure 8 that the friction coefficient of the composites first increases and then decreases with the increase in CeO_2_ content, while the wear rate of the composites first decreases and then increases with the increase in CeO_2_ content. When the CeO_2_ content is 0.6 wt%, the friction coefficient of the composite reached the maximum value of 0.32, and the wear rate reached the minimum value of 1.02 mg/m. The reason is that when the CeO_2_ content of composites is less than 0.6 wt%, with the increase in CeO_2_ content, the number of precipitated Si particles in the composites increases, and the size decreases gradually. In the process of friction, the number of hard particles bearing friction force per unit area increases, the force of single Si particle decreases, and the precipitated Si particles are not easy to fall off from the matrix, so the friction coefficient of the composites increases and the wear rate decreases gradually. When the content of CeO_2_ is more than 0.6 wt%, the size of precipitated Si particles in the composites increases, and the number of precipitated Si particles decreases gradually. The number of hard particles bearing friction force per unit area is less, and the precipitated Si particles are easier to fall off from the matrix because the force of the single Si particle becomes larger, therefore, the friction coefficient of the composites decreases and the wear rate increases gradually.

**Figure 9:**
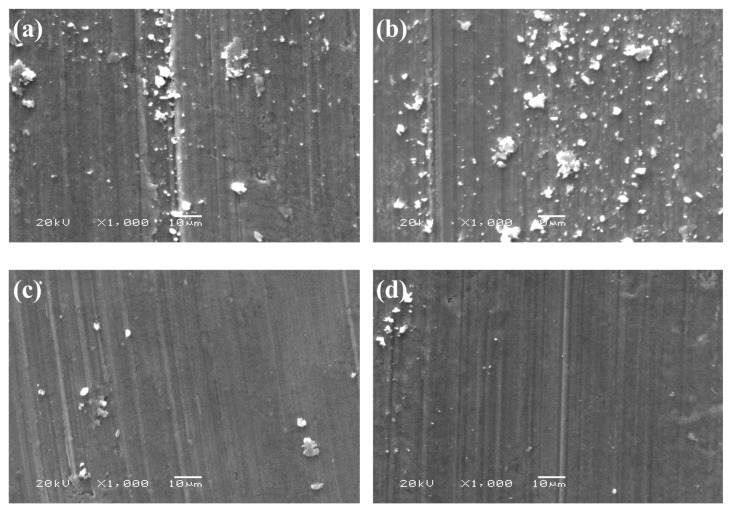

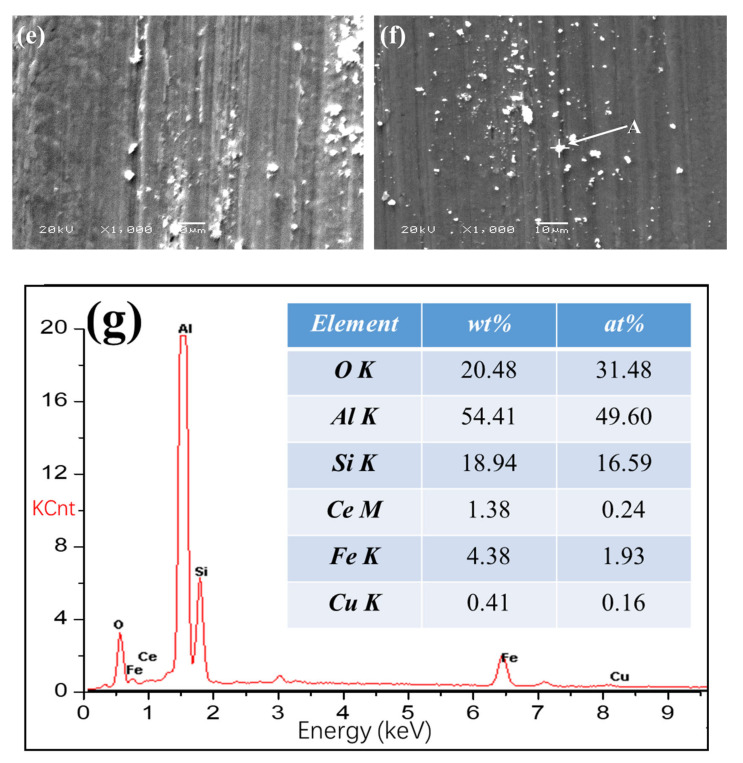
The SEM and EDS pictures of wear surface morphologies of SiCp/Al-Si composites with different CeO_2_ contents: (**a**) 0; (**b**) 0.15 wt%; (**c**) 0.30 wt%; (**d**) 0.60 wt%; (**e**) 1.20 wt%; (**f**) 1.80 wt%; (**g**) the energy spectrum diagram of point A in Figure 9f.

Figure 9 shows the SEM images and energy spectra of the wear surface morphologies of SiCp/Al-Si composites with different CeO_2_ Contents. Due to the complexity and randomness of the wear process, it is difficult to distinguish the types of hard particles attached to the worn surface. However, their types can be roughly determined by analyzing the energy spectrum. The results show that the wear surfaces of the composites have different degrees of furrow, and the matrix is oxidized. The corresponding oxides and precipitated silicon adhere to the wear surface. These furrows are the typical characteristics of abrasive wear. When the content of CeO_2_ is less than 0.6 wt%, with the increase in CeO_2_ content, the furrows on the wear surface become more and more regular, the width of furrows gradually narrowed and the number increased, and the damage phenomenon on the wear surface of the composite gradually reduced. When the content of CeO_2_ exceeds 0.6 wt%, with the increase in CeO_2_ content, the number of furrows on the worn surface decreases and the width increases, and the wear-out failure of the composite wear surface is more and more serious. The above phenomenon is mainly related to the size and quantity of Si particles precipitated in the composites. During the wear process, precipitated Si particles fall off, then mix between the friction pair and the wear surface under the action of load. As hard points, these Si particles continue to participate in the wear process and cause abrasive wear on the worn surface. When the content of CeO_2_ is less or more, the amount and size of precipitated Si particles in the composites are small and large, and the Si particles are easy to fall off during the wear process, so that a small number of large-sized Si particles cause serious damage to the wear surface. When the CeO_2_ content is around 0.6 wt%, the precipitated Si particles do not easily to fall off. However, a large number of Si particles fall off during the wear process. Because of their small size, the wear of these Si particles on the wear surface is more uniform, resulting in a large number of narrow furrows. Additionally, the wear surface is relatively smooth.

### 3.3. Effect of Load on Friction and Wear Properties of SiCp/Al-Si Composites with 0.6wt% CeO_2_

**Figure 10:**
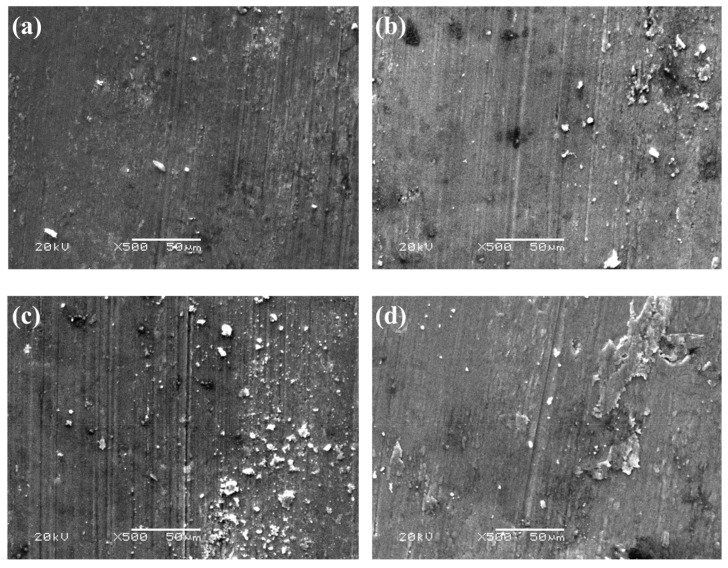

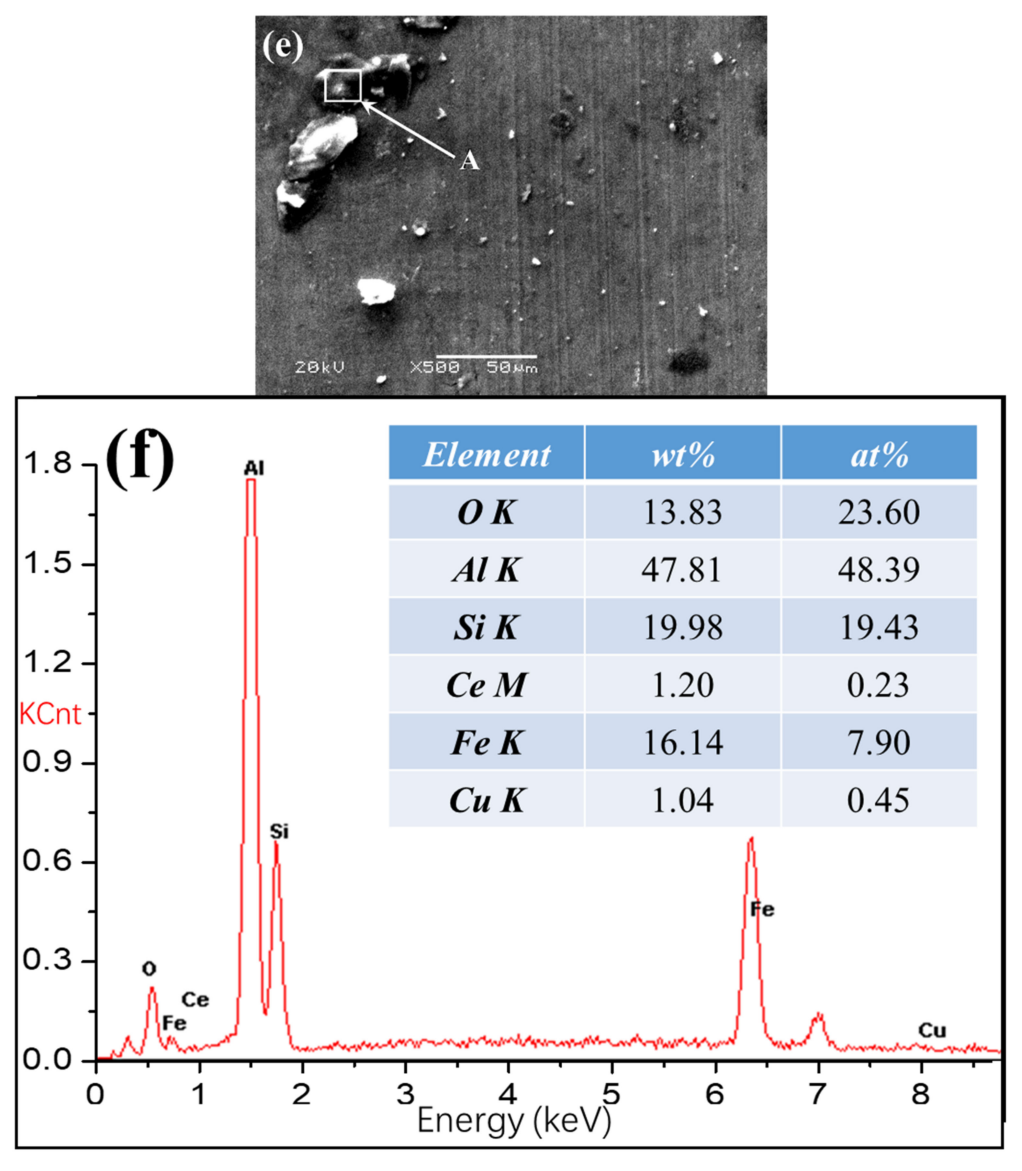
The SEM and EDS pictures of wear surface morphologies of SiCp/Al-Si Composites with 0.6 wt% CeO_2_ under different loads: (**a**) 500 N; (**b**) 550 N; (**c**) 600 N; (**d**) 650 N; (**e**) 700 N; (**f**) the energy spectrum diagram of region A in Figure 10e.

Figure 10 shows the SEM images and energy spectra of the wear surface morphologies of SiCp/Al-Si composites with 0.6 wt% CeO_2_ under the load of 500 N, 550 N, 600 N, 650 N and 700 N. It can be seen that when the load is 500 N, a large number of fine furrows are distributed on the wear surface, and a small amount of abrasive particles are distributed on the furrow surface. The separated abrasive particles further cause abrasive wear on the composite and accelerate the wear of the material. The wear mechanism of this process is mainly oxidation wear and abrasive wear. When the load is 550 N, the abrasive shedding phenomenon is more serious, there are more abrasive particles on the wear surface, and the furrow is obvious. The wear mechanism of this process is mainly abrasive wear. When the load is 600 N, a large number of abrasive particles are distributed on the wear surface, and the number of furrows on the wear surface increases, which leads to severe abrasive wear. When the load is 650 N, it can be seen that local plastic deformation occurs on the wear surface, and the damage of the wear surface is serious. The main wear mechanism of this process is adhesive wear. At a higher load of 700 N, the debris on the wear surface can be seen to be connected to a large block, which leads to serious adhesive wear.

**Figure 11:**
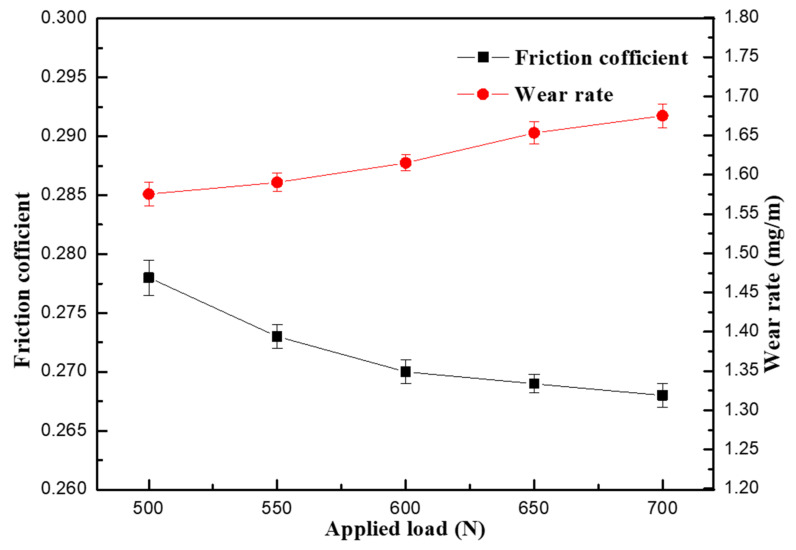
The curves of the friction coefficient and wear rate of SiCp/Al-Si composite with 0.6 wt% CeO_2_ content under different loads.

Figure 11 shows the curves of the friction coefficient and wear rate of SiCp/Al-Si composite with 0.6 wt% CeO_2_ content under different loads. It can be seen from Figure 11 that the friction coefficient of the composites first decreases with the increase in load while the wear rate slightly increases with the increase in load. The results are consistent with the microstructure analysis.

Under the action of periodic friction shear stress, the precipitated Si particles on the wear surface of the composites cannot be eliminated in time, which will lead to abrasive wear. Under the lower load, there are fewer abrasive particles falling off, and the wear damage degree of the composite surface caused by abrasive wear is lighter. With the increase in load, much more abrasive particles fall off, and the wear failure phenomenon of the composite surface is more obvious. When the load continues to increase, the heat generated in the friction process increases. When the temperature reaches a certain condition, local plastic deformation will appear on the worn surface, and the wear debris becomes soft and connected into blocks, resulting in serious adhesive wear.

## 4. Conclusions

The SiCp/Al-Si composites with different CeO_2_ contents were successfully prepared by a powder metallurgy method. The appropriate amount of CeO_2_ can obviously refine the size of precipitated Si particles. With the increase in CeO_2_ content from 0 to 1.8 wt%, the density, hardness, friction coefficient of the composites first increases and then decreases, the coefficient of thermal expansion (CTE) and wear rate of the composites first decreases and then increases.

When the content of CeO_2_ was 0.6 wt%, the composite showed the best comprehensive properties. The density and hardness of the composite reached the maximum value of 98.54% and 113.7 HBW, respectively. The CTE of the composite reached the minimum value of 11.1 × 10^−6^ K^−1^, the friction coefficient and wear rate of the composite reached the maximum value of 0.32 and the minimum value of 1.02 mg/m, respectively, when the load is 500 N.

CeO_2_ has little effect on the wear mechanism of the composites, and the wear mechanism of the composites with different CeO_2_ content is mainly abrasive wear under the load of 550 N. Compared with the content of CeO_2_, load has a great influence on the wear properties of the composites. The wear mechanism of the composites is mainly oxidation wear and abrasive wear under a low load. With the increase in load, the wear degree of abrasive particles is aggravated, and adhesive wear occurs under higher loads.
